# Unraveling uncertainties of water table slope assessment with DGPS in lowland floodplain wetlands

**DOI:** 10.1007/s10661-016-5642-3

**Published:** 2016-10-18

**Authors:** Dorota Mirosław-Świątek, Robert Michałowski, Sylwia Szporak-Wasilewska, Stefan Ignar, Mateusz Grygoruk

**Affiliations:** 1 Faculty of Civil and Environmental Engineering, Department of Hydraulic Engineering, Warsaw University of Life Sciences—SGGW, ul. Nowoursynowska 159, 02-776 Warsaw, Poland; 2 Faculty of Civil and Environmental Engineering, Water Centre Laboratory, Warsaw University of Life Sciences—SGGW, Ciszewskiego 6, 02-776 Warsaw, Poland

**Keywords:** Water level, Slope, DGPS, Elevation, Uncertainty, Wetlands, Measurements, Floodplain

## Abstract

In our study, we analyzed the combined standard uncertainty of water table slope assessment done using differential global positioning system (DGPS)-based measurements of water table elevation and distances between measurement locations. We compared and discussed uncertainties in water table slope assessments done in various hypothetical environments of lowland floodplains (water table slopes typically ranged from 1.25 · 10^−4^ to 1 · 10^−3^). Our analyses referred to elevation measurements done with the static GPS and DGPS real-time kinematic (RTK) approaches, which are currently among the most frequently used elevation measurement techniques worldwide. Calculations of the combined standard uncertainty of water table slope allowed us to conclude that the DGPS-RTK approach used in water table slope assessment can result in assessment errors as high as 50 % at short (<200 m) distances. Acceptable water table slope measurement errors (lower than 5 %) occur at distances longer than 11,320 m in the case of DGPS-RTK measurements, while, in the case of static GPS measurements, acceptable measurement errors at the same level occur at distances as low as 1350 m. Errors in water table slope assessment as high as 50 % occur at distances of 1130 m and 140 m for DGPS-RTK and static GPS measurements, respectively. We conclude that, although the DGPS-RTK methodology—due to its ease of use and time-saving capabilities is very often applied to water level measurements in lowland riparian wetlands, the application of the DGPS-RTK methodology for water table slope assessment at distances shorter than a few couples of meters results in very low accuracy (errors greater than 50 %) and should not be used for calculating local slopes in low slope areas such as lowland riparian zones.

## Introduction

Water level measurements have become a fundamental reference for any hydrological study. Such measurements are widely done for research and water management monitoring purposes worldwide (e.g., regular measurements on water gauges). Within frameworks of spontaneous monitoring of river stages, groundwater levels, or water table slopes calculations, water table elevations have always been crucial data for hydrological analyses. However, the quality of conceptually simple water table elevation measurements depends on the sites where the measurements are being done and on the methodologies applied. Due to the imperfection of measurement devices, the relativity of one’s senses and the changing physical conditions of the environmental background, measurement results—by definition—are always different from the original values measured. It is therefore essential to provide a quantitative evaluation of the quality of each measurement in order to be able to appropriately interpret the result of such measurement. Due to their relatively simplistic nature, uncertainties in water level estimation—contrary to well established analyses of hydrological modeling results (Beven 1993; Beven and Binley 1992), flood hazards (Dung et al. 2015) and water level-discharge relations (Bozzi et al. 2015; Le Coz 2012; Sikorska et al. 2013)—are seldom a subject of uncertainty analysis in hydrological literature. In the age of sophisticated models and integrated analyses of hydrological processes, quantitative analysis of the quality of fundamental water level measurements seems to have been forgotten. One may consider this surprising, given the rapid development of monitoring techniques allowing hydrologists to measure water levels with very high metric accuracy reaching decimals of millimeters and by conducting manual and automatic measurements in remote locations with harsh environments where, a decade ago, conducting hydrological measurements required a comprehensive set of optical devices (elevation leveler, total station), a number of indispensable pieces of equipment and extensive human resources and time.

Rapid development of remote satellite measurements using DGPS and increasing availability of measurement devices has allowed water level measurements to expand in recent years into sites where standard optical methods (water gauge-based and geodetic total station) had been very resource- and time-consuming, and hence had seldom been performed with the accuracy that would ensure quality (Popielarczyk 2012). This is mostly the case in wetlands, where unstable ground, frequent inundation and dense vegetation do not allow standard geodetic measurements to be widely done. Currently, because DGPS allows multiple elevation measurements to be done in a relatively short time, application of DGPS in wetlands has opened up a new field of research in wetland hydrology. Even though, when DGPS is used, a lower range of changes of a particular process, as well as a more significant influence of uncertainty on assessed variables, can be expected, DGPS-based water level measurements are indiscriminately done worldwide. Application of such measurements in remote wetland sites of broad and flat river valleys provides new data for two-dimensional hydraulic model calibration and validation. For practical reasons, such models and water table elevation measurements are used to analyze water table slopes, which are later used in determining flood extents, flood depths, and flood risk analysis. Surprisingly, although it is known that DGPS-based measurements are subject to certain errors, so far, not much has been done to address these errors in the field of water table slopes uncertainty assessment.

In our study, we assessed the combined standard uncertainty (*u*_*c*_) of water table slope (*S*_*w*_), which remains an indirect parameter that is assessed on the basis of direct measurements of water table elevation and distances between the measurement locations. Our study addresses the following questions: (1) what are the potential errors of water table slope assessment with the use of different DGPS-based water level and distance measurements?; (2) are DGPS-based measurements of water level reliable enough to be used in assessing local slopes?; and (3) what is the reasonable distance between water level measurement points for water table slope assessment in lowland floodplain systems that will allow DGPS-based measurements to be applied as a reliable compromise between the quality of measurements and the resources spent on measurement campaigns?

## Materials and methods

### Uncertainty of water level estimation

Independently from the measurement methodology, the real, physical value can never be accurately measured. The difference between the result of the measurement and the real value of the measured phenomenon is called an error of a measurement. Measurement errors are traditionally divided into gross errors (mistakes), accidental errors and systematic errors. A gross error usually results from distraction and carelessness of the person doing the measurement. This type of error may be limited to a minimum when measurement procedures are strictly followed but can hardly be totally avoided. Systematic errors result from imperfections of measurement devices and measurement methodologies. These errors can be limited by application of more and more accurate measurement methods and devices, but, like gross errors, can hardly be avoided. Accidental errors occur for multiple reasons, which usually cannot be foreseen and eventually avoided (e.g., sudden changes of temperature, movement of the air by the measurement device). Accidental errors are why each measurement of a particular phenomenon is unique. Due to the above definitions and the fact that the real value of a measured phenomenon is never known, the term “measurement error” is not used. Instead, along with the post-processing of particular measurements, the term “uncertainty” is used more frequently (JGCM 2008).

Uncertainty remains a parameter that is inherently combined with the result of a measurement and that describes a distribution of values, which, on a well-founded basis, can be assigned to the real value of a measured phenomenon. One of the main measures of the uncertainty of measurements is standard uncertainty, which is referred to in this paper as *u*.

In multiple cases, and in hydrology especially, particular values are not directly measured, but are indirectly assessed (calculated) as a function of the other values *x*_*i*_ that have been directly measured:

1 $$ y=f\left({x}_1,{x}_2\cdots, {x}_n\right) $$

which is referred to as an equation of a measurement. In such cases, uncertainty remains described as a combined standard uncertainty *u*_*c*_, and is calculated on the basis of the law of uncertainty propagation (Fuller 1987):

2 $$ {u}_c(y)=\sqrt{{{\displaystyle \sum_{i=1}^n\left(\frac{\partial f\left({x}_i\right)}{\partial {x}_i}\right)}}^2{u}^2\left({x}_i\right)} $$

where *u(x*_*i*_ *)* is the uncertainty of the value *x*_*i*_ that was directly measured.

### Assumptions of DGPS measurements

Accuracy of elevation and location measurements conducted using DGPS results from the fact that, with a particular measurement methodology (static/kinematic), systematic errors of measurements depend on the accuracy of the measurement device and on the distance between the measurement location and the reference station (Table 1).

**Table 1:** Standard uncertainties of water level *u(H)* and distance *u(L)* measurements done with different measurement techniques and measurement tackle

Measurement procedure	Source of corrections	u(H) [m]	u(L) [m]	Source
DGPS (GPS-RTK)	ASG EUPOS	0.03	0.05	www.asgeupos.pl
	Local network	0.010 + 1 ppm^a^	0.015 + 1 ppm^a^	TPI
Static GPS	ASG EUPOS	0.01–0.1	0.01–0.1	www.asgeupos.pl
	Local network	0.003 + 0.5 ppm^a^	0.005 + 0.5 ppm^a^	TPI

^a^ *ppm* parts per million—means that the error increases along with the increasing distance between the measurement device and the base station that is sending the correction

Both measurement methods analyzed, namely DGPS-RTK and static GPS, were developed to accurately measure a particular location’s coordinates, and both methods require at least one additional GPS device (a so-called base), which, during the entire time of measurement, is placed in the reference point of known XYZ coordinates. During the measurement, the base continually receives correction data. DGPS methodology assumes that the received corrections are processed in real time. Thus, corrections of the coordinates being measured are also generated in real time. Static measurements assume that the corrections are being stored and used for corrections of coordinate calculations in post-processing once the measurement is finished. The network of ASG-EUPOS consists of a network of reference stations distributed throughout Poland; correction data are widely available. The average distance between two neighboring reference stations in this network is approximately 70 km; these spatial distributions allow high accuracy in the corrections received. Corrections can either be imported from the nearest station of ASG-EUPOS network (which is highly applicable when measurements are being done a short distance from the reference station) or interpolated from a number of stations located in the neighborhood of the measurement location (a so-called areal correction) when measurements are being done at an approximately equal distance from a few reference stations. An individual reference station that is not interrelated with any of the networks must be placed at a point of known horizontal and vertical coordinates in order for it to be used in DGPS and static measurements.

Water table slope assessment requires elevation measurements of at least two points. The most important factor is a relative difference between measured elevations. Technical specification of particular measurement technologies (Table 1) allows the conclusion that the smallest errors in elevation measurements occur with the use of a local reference station and static measurements. The value of measurement errors increases because the DGPS-RTK measurements referred to the local reference station reach considerably higher uncertainties than the DGPS-RTK measurements referred to the national network of stations. The values of the errors (Table 1) allow the hypothesis that, in order to reach similar values of errors in water table slope assessment using DGPS-RTK methodologies referred both to a local reference station (more accurate) and a national network of stations (less accurate), the distances between measurement points should be increased considerably. One should also consider that, in order to use a local reference station in DGPS-RTK measurements, it is necessary to use two GPS devices at once. Moreover, in the case of real-time DGPS-RTK measurements, both GPS devices used must be equipped with radio transponders that allow internal communication between the measurement device and the base. In the case of static measurements, devices must be able to store large datasets referred to satellite signals. Static measurements due to their technical features and measurement methodology provide the most accurate GPS-based elevation surveys. Known disadvantages of static measurements are related to the fact that they require a long period (e.g., 30 min) of exposition at the monitoring point. During such a long measurement time, the water level can change, and the measurement is therefore likely to be inaccurate. By contrast, the use of a national network of reference bases in the RTK mode requires only one GPS device equipped with a GPRS modem that allows receipt of corrections via the GSM network. This method is therefore cheaper and less time-consuming and thus easier to apply under field conditions. Given the defined error values (Table 1), this method is likely to be applied in water table slope assessment on longer distances—uncertainties of results of slope assessment in the local scale (a few tens to hundreds of meters) are too large.

### Uncertainty assessment

**Fig. 1:**
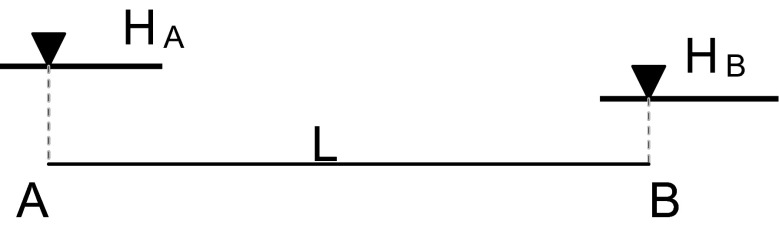
Direct absolute measured parameters used in water table slope assessment (*A* and *B*—two points located within the flooded plain; *H*_*A*_—water level in the point *A*; *H*_*B*_—water level in the point *B*; *L*—distance between the points *A* and *B*)

Assessment of the slope of a water table is done using an indirect approach on the basis of direct measurements of water level (*H*; [m a.s.l.]) and the distance (*L*; [m]) between the points where water level was measured (Fig. 1). Slope of the water table *(S*_*W*_ *)* is calculated according to the Formula 3.

3 $$ {S}_w=\frac{\left({H}_A-{H}_B\right)}{L} $$

Regarding the fact that the assessment of *S*_*W*_ remains an indirect estimation, the uncertainty of this assessment *u*_*c*_ *(S*_*W*_ *)* is calculated as a combined standard uncertainty using the law of uncertainty propagation (Formula 2). In this case, formula 2 is expressed as follows:

4 $$ {u}_c\left({S}_w\right)=\sqrt{{\left(\frac{\partial f\left({H}_A,{H}_B,L\right)}{\partial {H}_A}\right)}^2{u}^2\left({H}_A\right)+{\left(\frac{\partial f\left({H}_A,{H}_B,L\right)}{\partial {H}_B}\right)}^2{u}^2\left({H}_B\right)+{\left(\frac{\partial f\left({H}_A,{H}_B,L\right)}{\partial L}\right)}^2{u}^2(L)} $$

Function *f* presented in formula 2 is described by the measurement equation (Formula 3). After the determination of particular derivatives, Formula 4 may be presented as in Formula 5:

5 $$ {u}_c\left({S}_w\right)=\sqrt{2\cdot \frac{u{(H)}^2}{L^2}+{\left(\frac{H_A-{H}_B}{L}\right)}^2\cdot {\left(\frac{u(L)}{L}\right)}^2} $$

where *u(H)* is the uncertainty of water level measurement and *u(L)* is the uncertainty of distance measurement between points *A* and *B* (Table 1). Standard uncertainties given in Table 1 were assigned on the basis of the technical specifications of the instruments used for the measurements.

As the second element of Formula 5 expresses very low values, *u*_*c*_ *(S*_*w*_ *)* can be approximated by the simplified form (Formula 6). Hence, it appears that the uncertainty of the water table slope assessment within the flooded plain depends mainly on the uncertainty of the water level measurements and the uncertainty of the measured distance *L* between points A and B:

6 $$ {u}_c\left({S}_w\right)\approx \sqrt{2}\cdot \frac{u(H)}{L} $$

## Results

### Calculations

**Fig. 2:**
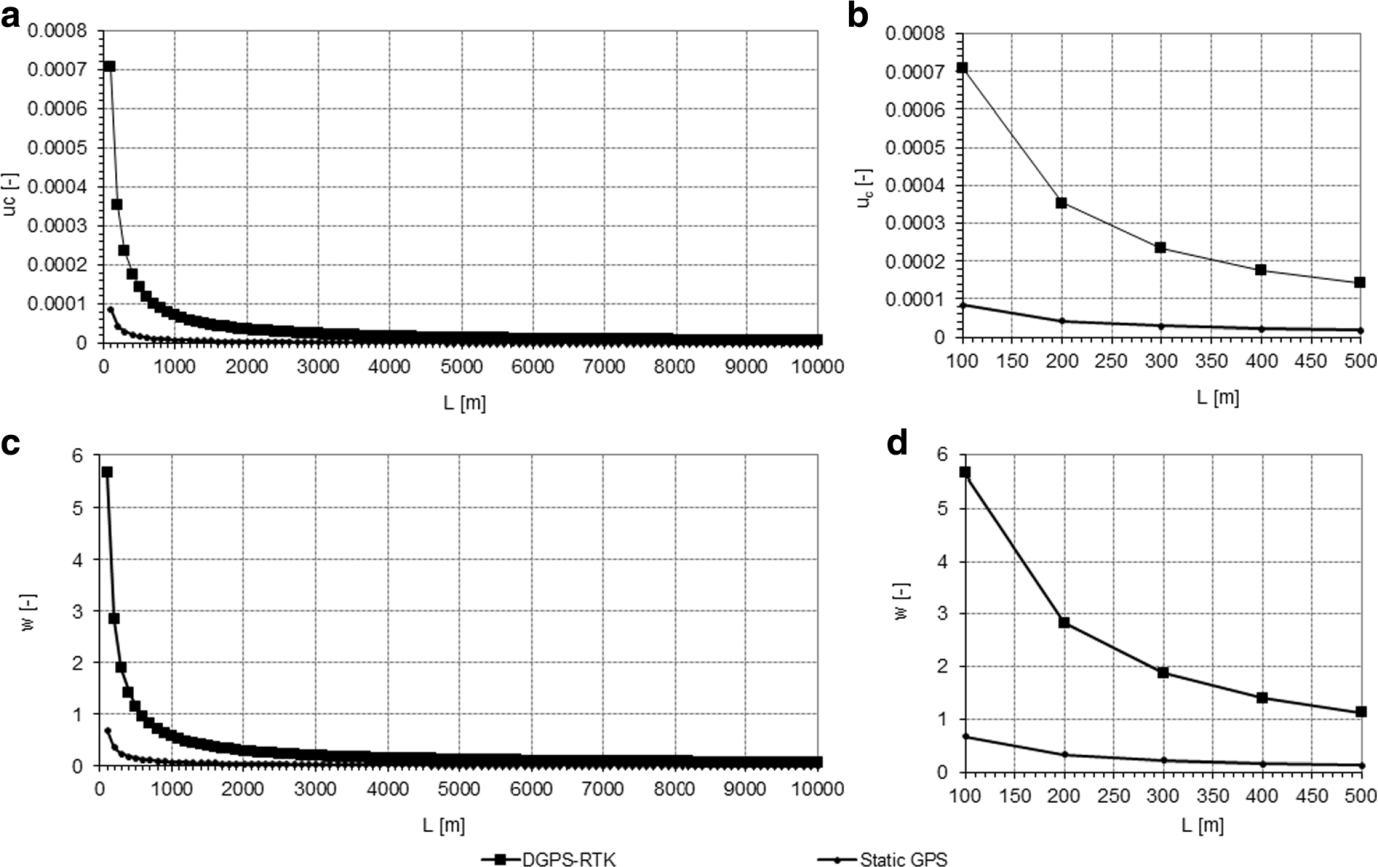
**a**, **b**—Relation between the distance between measurement points and uncertainty of water table slope assessment in the case of two GPS-based methodologies applied. (**a**—range of the distances 100–10,000 m; **b**—magnification of the **a** in the range of distances 100–500 m). **c**, **d**—Changes of the relative uncertainty of water table slope assessment (*w*) in the function of a distance *L* between measurement points. (**c**—range of the distances 100–10,000 m; **d**—magnification of **a** in the range of distances 100–500 m)

Formula 6 was applied in order to determine the influence of the distance on the uncertainty of water table slope assessment. Using two measurement technologies (DGPS-RTK and static GPS) and applying appropriate values of standard uncertainty *u(H)* (Table 1), the uncertainty of water table slope assessment was calculated as the function of the distance between the two hypothetical measurement points. We assumed that the distance *L* between two particular water level measurements done using DGPS-RTK and static GPS ranged from 100 m to 10 km. The results of the calculations revealed that the uncertainty of water table slope assessment decreases with the increasing distance between the points of water level measurements and strongly depends on the measurement methodology applied (Fig. 2).

The relation of standard uncertainties of particular measurements (DGPS*u*_*c*_ */*staticGPS*u*_*c*_) shows that, for a distance of 100 m, the *u*_*c*_ of the measurement done with the use of DGPS-RTK is more than eight times higher than the *u*_*c*_ of the measurement done with the use of static GPS measurement methodology, which measurement is as high as 8.33. Estimated values of combined standard uncertainty of water slope depend on the distance between the measurement points and standard uncertainties of measurement methodologies. Therefore, assessment of the influence of uncertainty on accuracy of water table slope is of crucial interest for processing of field-collected data. We assessed the relation of uncertainty to the estimated value (directly, through accuracy of the measurement) on the basis of the relative uncertainty *w(x)*:

7 $$ w(x)=\frac{u(x)}{x} $$

where *x* is the measured value.

In our approach, we considered the measured value analyzed in relation to the water table slope *S*_*w*_. It was assumed that the value of *S*_*w*_ equals 1.25 · 10^−4^, which is representative of water table slopes measured during the flood episode in the floodplain of the Biebrza Valley in March 2014. The value of *S*_*w*_ was obtained through precise geodetic measurements done with the use of a digital elevation leveler according to the methodology described in section 2.1. Moreover, the given values of *S*_*w*_ are similar to the ones obtained in modeling studies with the use of the one-dimensional river and floodplain flow model (Mirosław-Świątek et al. 2006).

**Fig. 3:**
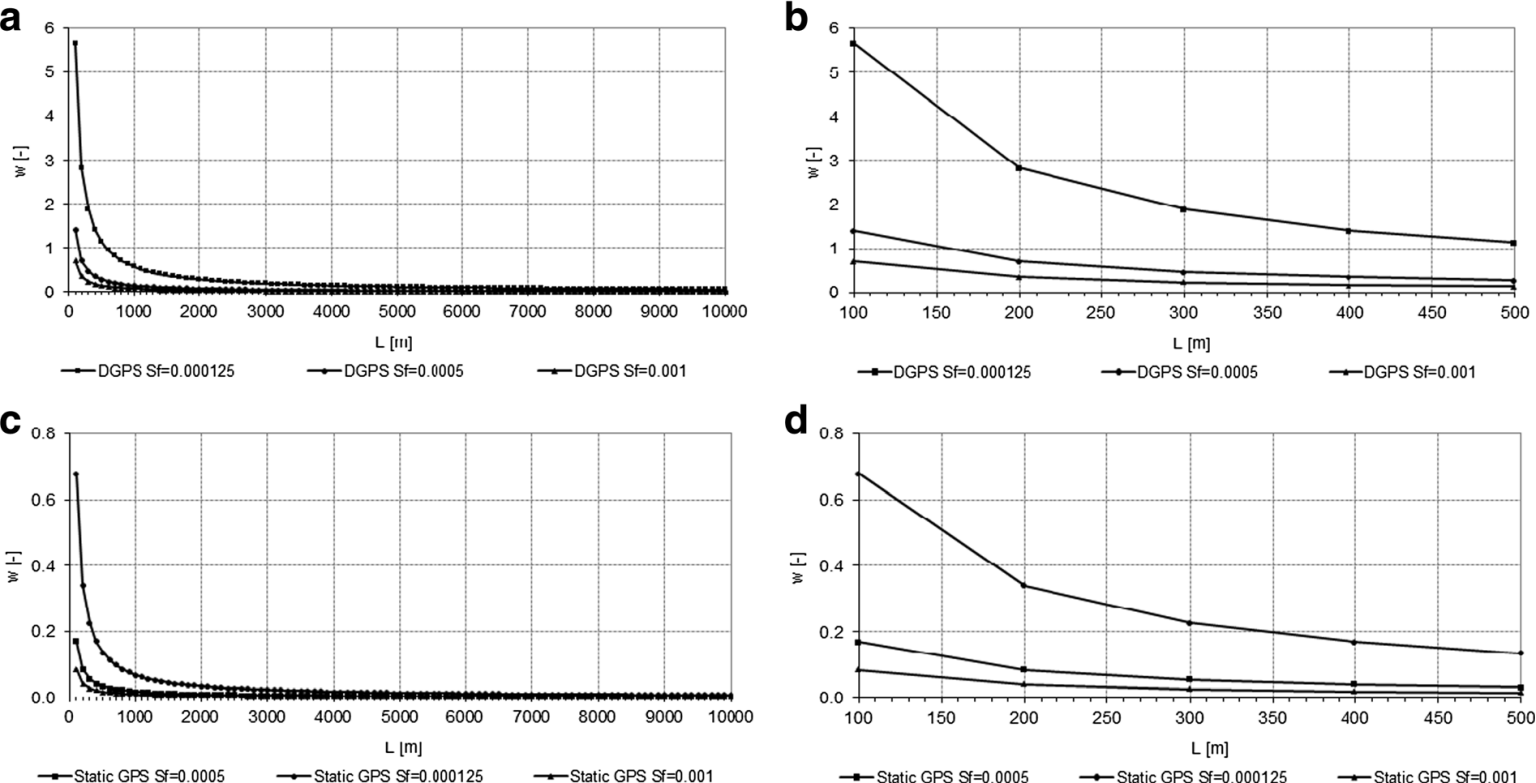
Changes of the relative uncertainty of water table slope assessment (*w*) in the function of a distance *L* between measurement points for various hypothetical environments of water table slope from 1.25·10–4 to 1·10–3. **a**—DGPS-RTK; **b**—close-up of **a** in the distances 100–500 m; **c**—static GPS; **d**—close-up of **c** in the distances 100–500 m

Changing values of *w* referred to the given slope of 1.25 · 10^−4^ and distances between measured water table elevations (*L*) are presented in Fig. 3. In the case of assumed distance *L* = 100 m, the accuracy of the DGPS-RTK-based slope measurement is approximately 5.5 times lower than the static GPS measurement. Measurement error in the case of static GPS reached 67 % in this case. In order to analyze the influence of changing slopes of water tables in different environments on the relative uncertainty of water table slope assessment based on DGPS-RTK elevation measurements, we analyzed exemplary cases of a typical lowland rivers (valleys), where *S*_*w*_ reached 1.25 · 10^−4^ through 5 · 10^−4^, up to 1 · 10^−3^ (Fig. 3a–d). We revealed that similar levels of *w* on short distances are reached for DGPS-RTK measurements in a river with an assumed slope of 1 · 10^−3^ and static GPS measurements in a river with an assumed slope of 1.25 · 10^−4^. These calculations allow us to state that DGPS-RTK measurements of water table elevation can only be considered representative and accurate enough for water table slope assessments of relatively steep (~1 · 10^−3^ ) slopes (i.e., in the cases of fast-running lowland rivers).

Calculated values of maximum errors of water table slope assessment and related critical distances between the measurement points are shown in Fig. 3a–d. Acceptable water table slope measurement errors (lower than 5 %) occur for distances longer than 11,320 m in the case of DGPS-RTK, while, in the case of static GPS measurements, acceptable values of measurement errors occur at distances as low as 1350 m. Errors in water table slope assessment as high as 50 % occur at distances of 1130 m and 140 m for DGPS-RTK and static GPS, respectively (Fig. 3).

### Field verification

**Fig. 4:**
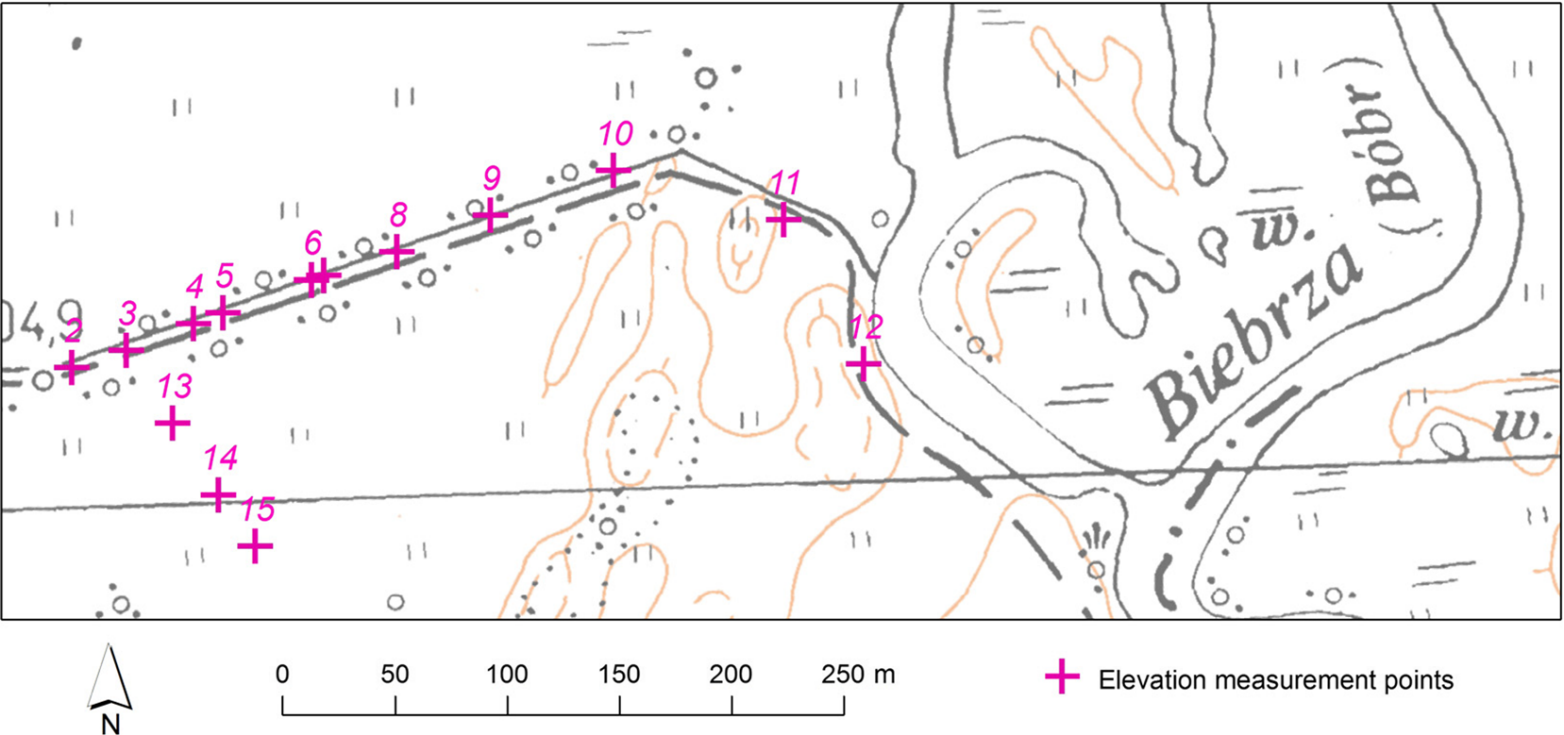
Field research-based comparison of water table slope measurement during flood. Northern reach of the lower basin of the Biebrza Valley, Poland. Locations of measurement points. Whole area presented on the map was flooded during the measurement campaign

In order to verify whether theoretical research is reflected in the field conditions, we conducted field measurement campaign in April 2014, during the spring thaw flood episode in the Biebrza Valley. Study area (Fig. 4.; GPS coordinates 53.42939^o^ N, 22.53014^o^ E) is located in the northern stretch of the lower basin of the Biebrza Valley, known for its unique environmental features related to flooding and floodplain management (Grygoruk et al. 2013; Chormański et al. 2009; Keizer et al. 2014; Mirosław-Świątek et al. 2016a; Świątek et al. 2008; Szporak et al. 2008). The processes of flooding and detailed analysis of both elevation and hydrological data conducted in this particular area indicated the need of computation-related flood parameter’s uncertainty analysis in a regional scale (Mirosław-Świątek et al. 2016a, Mirosław-Świątek et al. 2016b). Water table slopes were measured in two control transects: “2–12” (W-E-SE) of the total length of 410 m and “3–15” (NW-SE) of the total length reaching 105 m. During the measurement campaign, the whole area presented on the Fig. 4 was flooded and the average depths of water reached from 0.12 m (on the transect “2–12”) up to 0.55 m (in the points 14 and 15). Floods in the area analyzed are extremely low dynamic (e.g., no waves) and diurnal water level changes seldom exceed 0.01 m. Due to these reasons, the given stretch of the floodplain was selected to verify the hypotheses stated in this study.

**Fig. 5:**
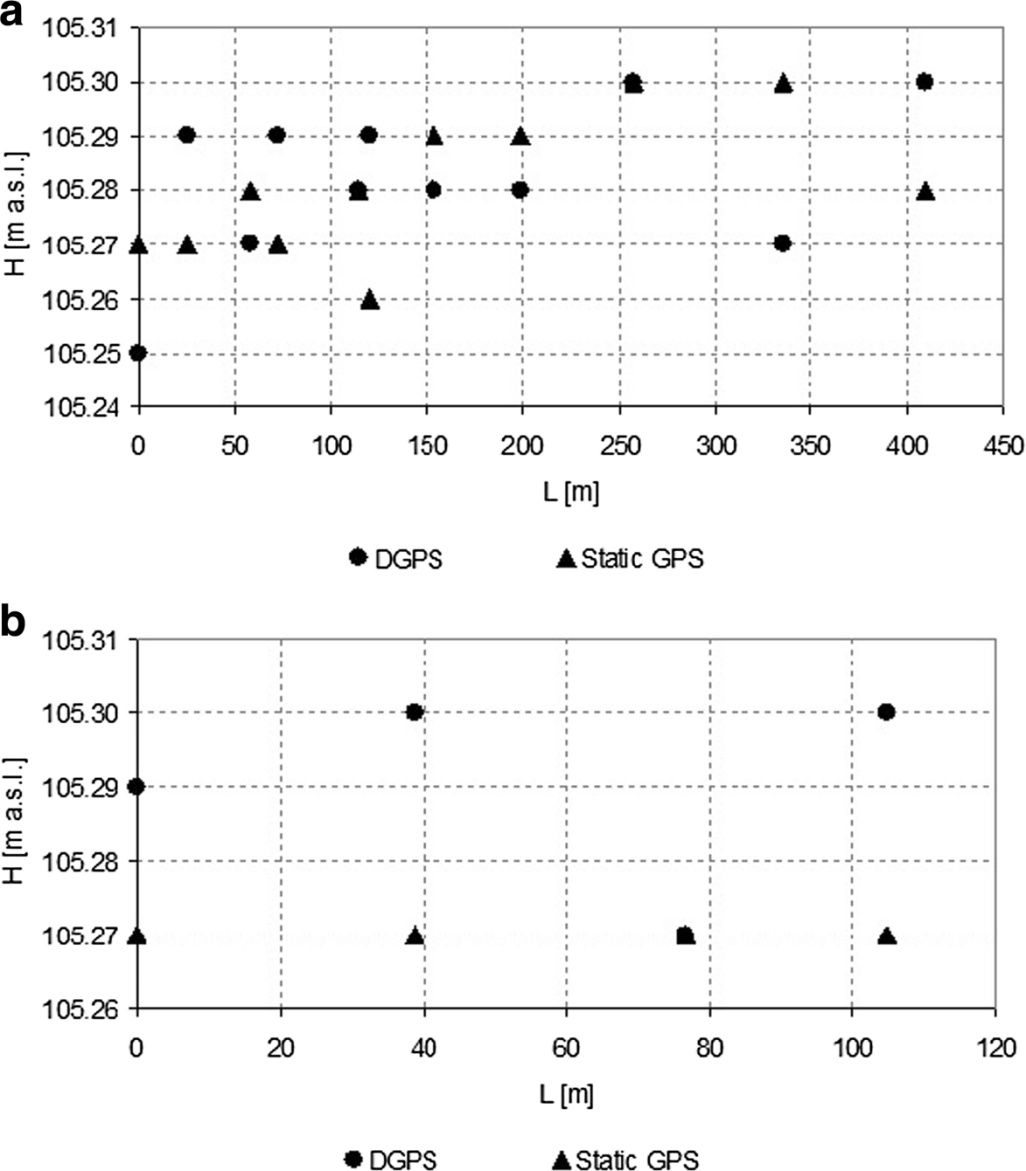
DGPS- and static GPS-measured water table elevations in the transect P2-P12 (**a**) and P3-P15 (**b**)

These transects allowed us to control the accuracy of water table slope assessment in different lengths representing the range of the most frequently used water table slope measurements distances. Differences between water table elevations measured with DGPS-RTK and static GPS varied up to 0.03 m (Fig. 5a, b). Measured water table elevations were applied to water table slope calculations along the two selected transects. Along the transect P2-P12 (Fig. 5a), the calculated *S*_*w*_ based on DGPS-RTK measurements reached as high as 0.00014, being nearly five times higher than the *S*_*w*_ assessed on the basis of static GPS measurements of water table elevation. Such a difference fits to the theoretically calculated *u*_*c*_ for both measurement techniques analyzed (Fig. 2b), at the given distance *L* between two elevation measurements reaching as high as 300 m. Along the transect P3-P15 (Fig. 5b), water table elevation measurements done with the use of static GPS indicate that the *S*_*w*_ at the measured distance of 105 m is equal to 0. Contradictory—the use of DGPS-RTK methodology to water table slope assessment resulted in the *S*_*w*_ value as high as 2.86 · 10^−4^ (locally, at shorter distances DGPS-RTK-assessed *S*_*w*_ reached 1 · 10^−3^ which is unusual in the reality of floodplain analyzed).

## Discussion

Although the DGPS-RTK methodology—due to its ease of use and time-saving capabilities—is very often applied in water level measurement in wetlands, its application to water table slope assessment at distances shorter than a few couples of meters results in very low accuracy (errors greater than 50 %). Water table slope assessment is considerably more accurate when static GPS measurement methodology is applied. This methodology reduces possible maximum errors to lower than 10 % at a distance as low as 650 m. In the case of static GPS measurements, however, both the duration of a single measurement and post-processing of measured data and corrections are longer and more complicated than in the case of DGPS-RTK.

**Fig. 6:**
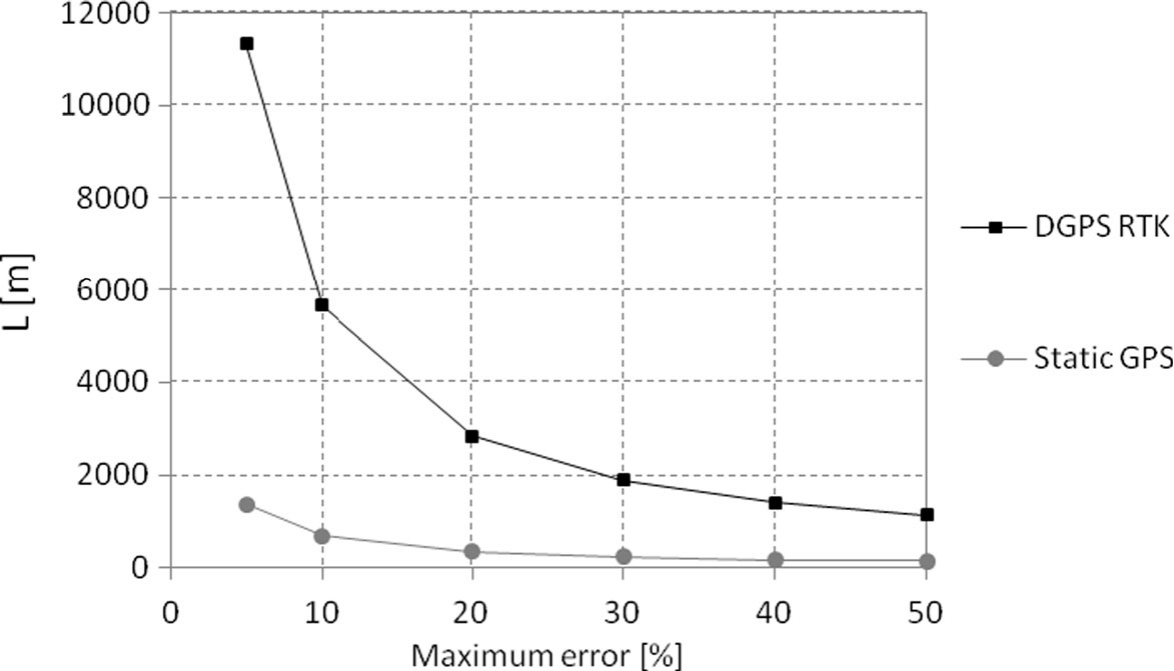
Maximum error of water table slope assessment with DGPS-RTK and static GPS applied in various distances *L* between the measurement points

Intuitively, one might expect that water table slope assessment using DGPS-RTK at distances of 1000 m should not generate excessive errors. On the contrary, as shown above, errors in measurement can be as high as 56 % when this methodology is used (Fig. 3a; Fig. 4). One should also consider the fact that calculated values of relative uncertainty and maximum errors were estimated for the known value of a slope reaching 1.25 · 10^−4^: an average slope of a typical lowland floodplain. However, the local values of water table slopes can range from 5 · 10^−5^ to 1 · 10^−4^. Therefore, measurements of water table slope that are lower than the representative value of 1.25 · 10^−4^ may entail an even greater percentage of error than that shown in Fig. 6.

For instance, if the reference water table slope reaches 1 · 10^−3^, then, given the technical specifications of the DGPS-RTK measurements of water table slope at a distance of 1000 m, the maximum error percentage will decrease by approximately eight times, compared to the reference slope of 1.25 · 10^−4^, and will reach only 7 %. Conversely, if the reference slope of a water table is smaller, reaching only 1 · 10^−4^, then the maximum error percentage will increase and, at a distance of 1000 m, will reach approximately 71 % if measured with the use of the DGPS-RTK methodology. Similarly, a high error percentage can occur in cases of spatial extrapolation of DGPS-RTK slope assessment (e.g., in situations in which measurement in a location 500 m from the measurement point is not possible due to inaccessibility caused by high water level or extremely dense vegetation). In such conditions, if the reference slope in the area reaches the assumed value of 1.25 · 10^−4^, the error of slope assessment at a distance of 1000 m can reach 56 %, which, in extreme conditions, may result in the calculated value of the water table slope reaching 1.96 · 10^−4^. If this calculated value of the water table slope is used to determine water level in a remote location which is, for example, 500 m downstream from the last measurement, the accuracy of water level estimation at this point will reach 0.11 m. Such a value in a situation in which water depth in the flooded plain reaches 0.5 m entails errors in water depth assessment as high as nearly 21 %. Therefore, results presented in our paper are important not only for studies aimed at water table slope assessment, but also for studies of spatially interpolated flood depths, validation of river bathymetry (Skinner 2009), river and floodplain morphodynamics (Eekhout et al. 2014), and quantitative assessment for water exchange in natural, lowland rivers during flood episodes (Harrison et al. 2012), which is relevant in flood risk management and habitat analyses for certain valuable species (Maciorowski et al. 2014). We also suspect that our findings could have considerable importance monitoring of other types of water-related ecosystems, such as lakes, estuaries, and the sea (Ngagipar and Yusof 2011). Presented results can also be extended to the use of DGPS-RTK technology as a tool in elevation assessment of monitoring points, where accurate surface water and groundwater elevations are a subject of detailed hydrological analyses based on datasets of high spatial and temporal accuracy, including field data-based model calibrations (Świątek et al. 2008; Grygoruk et al. 2014; Booth and Loheide 2012).

In harsh field conditions that include unstable ground (peat soil), flooding, long walking distances, and inaccessibility due to the presence of dense wetland vegetation, the use of standard geodetic measurement devices becomes challenging. Thus, currently, elevation measurements are predominantly done using remote satellite techniques such as DGPS-RTK and static GPS. Although the accuracy of a single DGPS-RTK measurement of water table elevation reaching 0.05 m may be sufficient for general hydrological analyses, it is important to recognize that the application of this methodology to water table slope assessments within large lowland floodplains includes vast uncertainty and errors that may distort the final results of hydrological analyses (e.g., calibration of two-dimensional hydrodynamic models).

## Conclusions

Our theoretical study, which was based on the real field conditions of a lowland floodplain wetland during a flood, revealed that the application of DGPS-RTK elevation measurement technology for water table slope assessment may potentially generate significant errors (Szporak-Wasilewska et al. 2015). Assessment of the water table slope at the distance of 100 m using the DGPS-RTK methodology for water level measurements may potentially result in exaggeration of the assessed water table slope compared to the real water table slope by a factor of 5.6. Errors in water table slope assessment using DGPS-RTK methodology are reduced to approximately 6 % when the water level measurement points are separated by approximately 10,000 m. Considerably fewer errors in water table slope assessment occur when static GPS measurements are used. In such cases, when the two points used for water table slope analysis are separated by 100 m and 1000 m, the maximum errors in water table slope assessment can be 67 % and 7 %, respectively. When the water level measurement points are separated by 400 m, errors in water table slope assessment can be approximately 17 %.

Our conclusions are especially important at this time, when satellite-based elevation measurements have overtaken optical measurements. Although the DGPS methodology is being widely applied in water level and water table slope assessments worldwide due to its ease of use and low demands on time and human resources, we strongly recommend (if field conditions only allow) that the use of either static GPS or classical optical measurements be considered, especially in cases in which water tables have low slopes during floods in lowland riparian geoecosystems.
